# An adaptive multiscale local mesh ternary pattern technique with extensive pre-processing and Grey Wolf optimisation based classifiers for oral cancer image classification

**DOI:** 10.1038/s41598-026-47931-7

**Published:** 2026-04-09

**Authors:** Varun Srivastava, Khushi Garg, Samarth Soni, Arun Balodi, Manoj Tolani, Vikash Singh

**Affiliations:** 1 Department of Computer Science and Information Technology, Jaypee Institute of Information Technology, Noida, Uttar Pradesh 201309 India; 2 Department of Electronics and Communication Engineering, Dayananda Sagar University, Karnataka 562112 Bengaluru, India; 3 Department of Electronics and Communication Engineering, Jaypee Institute of Information Technology, Noida, Uttar Pradesh 201309 India; 4 Manipal Institute of Technology, Manipal Academy of Higher Education, Manipal, India

**Keywords:** Biomedical image processing, Machine learning, Texture-based feature extraction, Deep learning, Image Processing, Cancer, Computational biology and bioinformatics, Engineering, Mathematics and computing

## Abstract

This study introduces an enhanced texture-based algorithm for the classification of oral cancer images. The images are first extensively preprocessed to enhance the affected area with techniques like gamma correction, adaptive histogram equalization, and sharpening of images using a Laplacian filter. Then a feature descriptor is extracted using an Adaptive Multiscale Local Mesh ternary patterns, which uses an adaptive threshold, and a sliding window of multiple scales. A machine learning model is then used for classification, which is also optimised using Grey Wolf optimisation. The pedagogy yields an overall average accuracy of 98.29% and 99.89% on two publicly available datasets. Also, the model is compared to three state-of-the-art techniques for oral cancer detection and is found to give an average improvement of 9.21% and 20.67% on the respective datasets.

## Introduction

Oral cancer cases are continuously increasing, causing a challenge within the domain of oncology, and it’s becoming a global challenge that needs an early prediction for effective treatment^1,2^. Among the various diagnostic modalities available for oral cancer, non-invasive imaging techniques have emerged as valuable tools for early detection and assessment^3,4^. This paper aims to develop a texture feature-based algorithm for the detection of oral cancer, enhancing the identification and characterization of oral tissue abnormalities. 

In recent years, there has been a surge in the application of Artificial Intelligence (AI) and Machine Learning (ML) techniques in medical imaging for oral cancer diagnosis. Deep learning-based models, particularly Convolutional Neural Networks (CNNs), have demonstrated substantial progress in histopathological and photographic image classification^5–7^. For instance, Devindi et al.^7^ proposed a multimodal deep CNN pipeline integrating both imaging and patient metadata for early oral cancer detection, achieving 81% accuracy, while Das et al.^5^ introduced an ensemble deep learning framework for Oral Squamous Cell Carcinoma (OSCC) detection that attained 97.88% accuracy. Similarly, Goswami et al.^8^ utilized Light Gradient Boosting Machine (LightGBM) with color and texture features to classify pre-cancerous oral stages with over 99% accuracy. Despite these promising outcomes, deep models often demand large datasets, high computational resources, and complex tuning strategies, making them less practical in resource-limited healthcare environments. Therefore, robust handcrafted feature-based algorithms remain essential for efficient, interpretable, and low-cost diagnostic systems.

Similar Deep learning based frameworks are also developed for related datasets of the mouth. For example, deep learning-based optimal feature extraction has been applied for ulcer classification in wireless capsule endoscopy (WCE) images^9^. Explainable AI frameworks have been developed for localization and classification of gastrointestinal tract disorders from endoscopic images^10^, emphasizing the importance of transparency in medical decision support. In oral cancer research, transformer-based models and few-shot learning strategies have been explored for histopathological image classification^11^, while personalized transfer learning CNN architectures have been proposed to enhance oral cancer detection performance^12^. These studies demonstrate the strong potential of deep learning, however, they also underscore challenges related to data requirements, interoperability, and computational complexity, thereby motivating the development of lightweight and adaptive texture-based frameworks such as the proposed AMLMTP approach. The texture-based methods such as Local Binary Patterns (LBP) and Local Mesh Patterns (LMeP) have shown potential in analyzing the microstructural irregularities of oral tissues^13,14^. However, conventional texture descriptors are often limited by their sensitivity to illumination, scale, and noise variations. Recent studies have addressed these limitations through adaptive multiscale analysis, local feature fusion, and intelligent optimization-based classification^15–17^. For example, Huang et al.^15^ proposed an intelligent multisampling tensor model for oral cancer MRI image classification using fusion strategies, while Xu et al.^17^ developed a multiscale vision transformer framework (MedTrans) for Oral Squamous Cell Carcinoma detection, outperforming CNN-based architectures. Motivated by these advances, the present work introduces an Adaptive Multiscale Local Mesh Ternary Pattern (AMLMTP) framework coupled with extensive preprocessing and Grey Wolf Optimisation (GWO)-based classifier tuning. This hybrid approach aims to enhance diagnostic precision while maintaining computational efficiency and interpretability, making it suitable for both research and clinical applications.

Oral cancer is characterized by abnormalities in the oral cavity. These abnormalities can manifest as lumps, sores, or unexplained bleeding, and if not detected at the early stage, they can lead to severe complications^18,19^. These techniques offer a less intrusive alternative to traditional biopsy methods, which reduces the patient’s discomfort and the risk of complications^13,14,20,21^. The integration of a texture-based automated detection mechanism for image analysis helps to accelerate the monitoring of disease progression, allowing timely adjustments to control cancer spread^22,23^.

The novelty of this research lies in the introduction of an adaptive and multiscale enhancement to the conventional Local Mesh Ternary Pattern (LMTP) descriptor. The adaptive thresholding mechanism, combined with multiscale feature extraction, enables the proposed model to capture texture variations more effectively across multiple resolutions and illumination conditions. To ensure the input images provide the most discriminative information, a robust preprocessing framework incorporating image sharpening, gamma correction, and adaptive histogram equalization is employed, leading to significant improvement in image clarity and lesion visibility. Furthermore, machine learning classifiers including Support Vector Machine (SVM), Random Forest, and Light Gradient Boosting Machine (LightGBM) are optimized using the Grey Wolf Optimization (GWO) algorithm, resulting in improved accuracy and robustness through adaptive parameter tuning. The proposed framework is lightweight and interpretable compared to complex deep learning architectures, making it more accessible for clinical applications where computational resources are limited. Finally, comprehensive comparisons with state-of-the-art methods, including CNN ensembles^5^, multimodal pipelines^7^, and transformer-based architectures^17^, demonstrate that the proposed AMLMTP-GWO method achieves competitive or superior performance with reduced architectural complexity.

The integration of a texture-based automated detection mechanism for image analysis helps to accelerate the monitoring of disease progression, allowing timely adjustments to control cancer spread^22,23^. The following objectives are met while implementing the proposed algorithm:

- The already existing Local Mesh ternary pattern algorithm is significantly improved by introducing an adaptive threshold and multiscale feature extraction
- An advanced framework for preprocessing is presented, which is based on techniques like Image sharpening, Gamma Correction, and Adaptive histogram equalization, so that the image quality is highly increased along with improved visibility of the affected area.
- The ML models are then optimized using the Gray-Wolf optimization for improved accuracy. Different strategies of optimization are applied for different ML algorithms.
- The technique is also compared to other state-of-the-art techniques based on deep learning pedagogies and is found to yield better results with a less complex framework.

The paper is organized in the following manner. Section Literature review summarizes the various techniques used for oral cancer diagnosis. Section Methodology explains the proposed methodology step-by-step, and Sect. Results highlights the results achieved by using the proposed method. Finally, Sect. Conclusion concludes the final findings of the present research work.

## Literature review

Many researchers have presented various texture and shape based features for the classification of oral cancer images. Convolutional Neural Networks (CNNs) play a significant role in the classification in some of the proposed work. The authors of Ref. 24 developed a system for oral cancer detection using image enhancement, Marker Controlled Watershed Segmentation, and feature extraction, achieving 96% accuracy with GLCM and SVM. This study highlights the importance of precise methodologies in global oral cancer classification. The authors in Ref. 25 leveraged deep neural networks and transfer learning to improve early detection and diagnosis of oral cancer. Among the various algorithms tested, Inception-V3 demonstrated superior accuracy in identifying intricate patterns associated with the disease. The authors in Ref. 26 used machine learning to predict outcomes in 467 OSCC (Oral Squamous Cell Carcinoma) patients, finding that decision trees achieved 70.59% accuracy and high specificity. This work underscores the potential of AI in improving treatment plans and follow-up strategies for OSCC. The authors in Ref. 27 reviewed the use of machine learning in diagnosing oral cancer, potentially malignant disorders, emphasizing early detection to improve prognosis. This study introduces AI concepts to help oral medicine scientists develop clinical detection models.

The authors of Ref. 28 explored the role of machine learning, particularly deep learning, in the early detection of oral cancer, emphasizing its effectiveness in identifying early lesions. In Ref. 29, a novel method for early oral cancer diagnosis using an optimized CNN was introduced, enhanced by the Seagull and Particle Swarm Optimization Algorithms. This method achieved superior accuracy (96.94%), precision (94.65%), recall (91.60%), and F1-score (88.55%) compared to other methods, highlighting its efficacy in detecting oral cancer. The authors in Ref. 30 highlighted the potential of optical imaging and AI-based approaches to improve the early diagnosis of Oral and OroPharyngeal Squamous Cell Carcinoma (OPSCC). The authors in Ref. 31 studied various AI methods, including deep learning, machine learning, fuzzy computing, data mining, and genetic algorithms, highlighting their applications, benefits, and drawbacks in the detection of oral cancer. The authors in Ref. 32 introduced a smartphone-based imaging method using HRNet deep learning to enhance oral cancer detection, achieving 83.0% sensitivity and 96.6% specificity. This study demonstrates the potential of centered image capture and resampling techniques in improving early diagnosis and survival rates. The authors in Ref. 33 introduced a deep learning algorithm using smartphone photos for oral disease diagnosis, achieving 84.3% accuracy and 83.6% F1 score. Their “center positioning” method and resampling techniques significantly enhanced diagnostic performance.

**Table 1:** Some major milestones in the field of oral cancer classification along with their limitations.

S. no.	Authors	Methodology	Results	Limitations
1	Chu et al.^26^	Applied machine learning to predict outcomes in OSCC patients.	Decision trees achieved 70.59% accuracy and high specificity.	The accuracy achieved was moderate.
2	De et al.^27^	Reviewed the use of machine learning in diagnosing oral potentially malignant disorders.	Emphasized the importance of early detection to improve prognosis.	The study lacks practical implementation details.
3	Huang et al.^29^	Used an optimized CNN with Seagull and Particle Swarm Optimization Algorithms.	Achieved 96.94% accuracy with high precision.	The optimization process is complex.
4	Lin et al.^32^	Used a smartphone-based imaging method with HRNet deep learning.	Achieved 83.0% sensitivity and 96.6% specificity.	The method is limited by the quality of smartphone images.
5	Mira et al.^33^	Introduced a deep learning algorithm using smartphone photos.	Achieved 84.3% accuracy and 83.6% F1 score with their proposed model.	The results depend heavily on photo quality and positioning.
6	Warin et al.^34^	Reviewed deep learning methods in diagnosing and predicting the prognosis of oral cancer.	Accuracy ranged from 85.0% to 100%.	varying accuracy across different studies.

In Ref. 35, the effectiveness of non-invasive fluorescence visualization for oral cancer screening was demonstrated, achieving 98.0% sensitivity but 43.2% specificity.

In Ref. 36, oral cancer detection using CNNs with 700 clinical images was addressed. DenseNet121 achieved 99% precision, 100% recall, and 99% F1 score, while faster R-CNN showed 76.67% precision, 82.14% recall, and 79.31% F1 score, highlighting their effectiveness in lesion classification and detection. In Ref. 34, the authors examined the use of deep learning in diagnosing and predicting the prognosis of oral cancer, covering studies from 2000 to 2023. Fifty-four studies were included, showing deep learning models with accuracy ranging from 85.0% to 100%. Table 1 highlights major contributions in the field of oral cancer classification along with some of their limitations.

Several studies have explored artificial intelligence (AI) and machine learning (ML) techniques for automated detection, classification, and segmentation of oral cancer lesions from various imaging modalities. Table 2 presents a comparative analysis of recent approaches, highlighting their methodologies, data modalities, strengths, and limitations.

The proposed methodology effectively addresses several challenges identified in the literature on oral cancer classification. Using two publicly available datasets and employing optimized machine learning models, predictive performance is improved. The proposed work overcomes limitations related to photo quality and positioning by integrating robust data handling and feature extraction techniques.

## Methodology

The following steps are used to classify the oral cancer images obtained from publicly available datasets.

### Preprocessing

The important features necessary for effective feature extraction becomes highlighted after using an extensive pre-processing technique. Following subsections describe the preprocessing steps used in the framework.

#### Contrast limited adaptive histogram equalization (CLAHE)

In this study, CLAHE is applied to enhance the contrast of oral cancer images by operating on small regions (tiles) rather than the entire image. It redistributes intensity values locally, improving contrast in areas with poor illumination and preventing the over-amplification of noise through contrast limiting.

**Table 2:** Comparative analysis of related oral cancer detection methods.

Study/method	Year	Technique used	Modality	Feature type	Optimization/classifier	Computational cost	Interpretability
Das et al.^5^	2024	Ensemble Deep CNN	Histopathology	Deep features	Softmax Ensemble	High	Medium
Devindi et al.^7^	2024	Multimodal CNN Pipeline	Oral Images + Metadata	Deep features	CNN + Metadata Fusion	High	Low
Xu et al.^17^	2024	Vision Transformer (MedTrans)	Histopathology	Attention-based deep features	Transformer Classifier	Very High	Medium
Goswami et al.^8^	2023	LightGBM + Color/Texture Features	Oral Images	Handcrafted (Color + Texture)	LightGBM	Low	High
Huang et al.^15^	2022	Intelligent Tensor Model (ITMO)	MRI Images	Fused texture features	Fuzzy Neural Network	Moderate	High
Balodi et al.^13^	2016	Local Mesh Pattern (LMeP)	Oral Tissue Images	Texture features	SVM	Low	High
Balodi et al.^14^	2024	Enhanced LBP-based Texture Analysis	Oral Images	Texture features	Random Forest	Low	High
Yaduvanshi et al.^3^	2024	CNN + Transfer Learning (VGG16)	Oral Photographic Images	Deep features	Softmax	High	Low
Kim et al.^1^	2019	Deep CNN for Oral Lesion Detection	Clinical Photos	Deep features	CNN	High	Low
Proposed AMLMTP-GWO	2025	Adaptive Multiscale LMTP + GWO Optimization	Oral Images	Adaptive Texture Features	Optimized SVM / RF / LightGBM	Low	Very High

Feature Type - indicates whether features are handcrafted or deep-learned; Computational Cost - based on model complexity and resource usage; Interpretability - qualitative indication of how explainable the model decisions are

The mathematical formulation of CLAHE is given by

1 $$\begin{aligned} J_{\text {CLAHE}}(x, y) = \frac{J(x, y) - J_{\text {min}}}{J_{\text {max}} - J_{\text {min}}} \cdot (L - 1) \end{aligned}$$

where $J_{\text {CLAHE}}(x, y)$ is the enhanced pixel intensity at position $(x, y)$, and $J(x, y)$ is the original pixel intensity. Similarly, $J_{\text {min}}$ and $J_{\text {max}}$ are the minimum and maximum intensity values in the local region, and $L$ is the number of intensity levels in the image.

#### Gamma correction

It is done to achieve a balance of the optimal brightness of the image, where some pixel areas are too dark or too bright.

The equation for gamma correction is expressed as

2 $$\begin{aligned} J_{\text {gamma}}(x, y) = \left( \frac{J(x, y)}{N} \right) ^\gamma \cdot N \end{aligned},$$

where $J_{\text {gamma}}(x, y)$ is the pixel intensity after gamma correction, $J(x, y)$ is the original pixel intensity, $N$ represents the maximum intensity value (typically 255), and $\gamma$ is the gamma correction factor. Values of $\gamma < 1$ brighten the image, while $\gamma > 1$ darken it. Here, the value of 0.4 is used, which yielded optimal brightness.

#### Image sharpening

A Laplacian filter with order 2 is then applied to extract the regions of rapid intensity change.

**Fig. 1:**
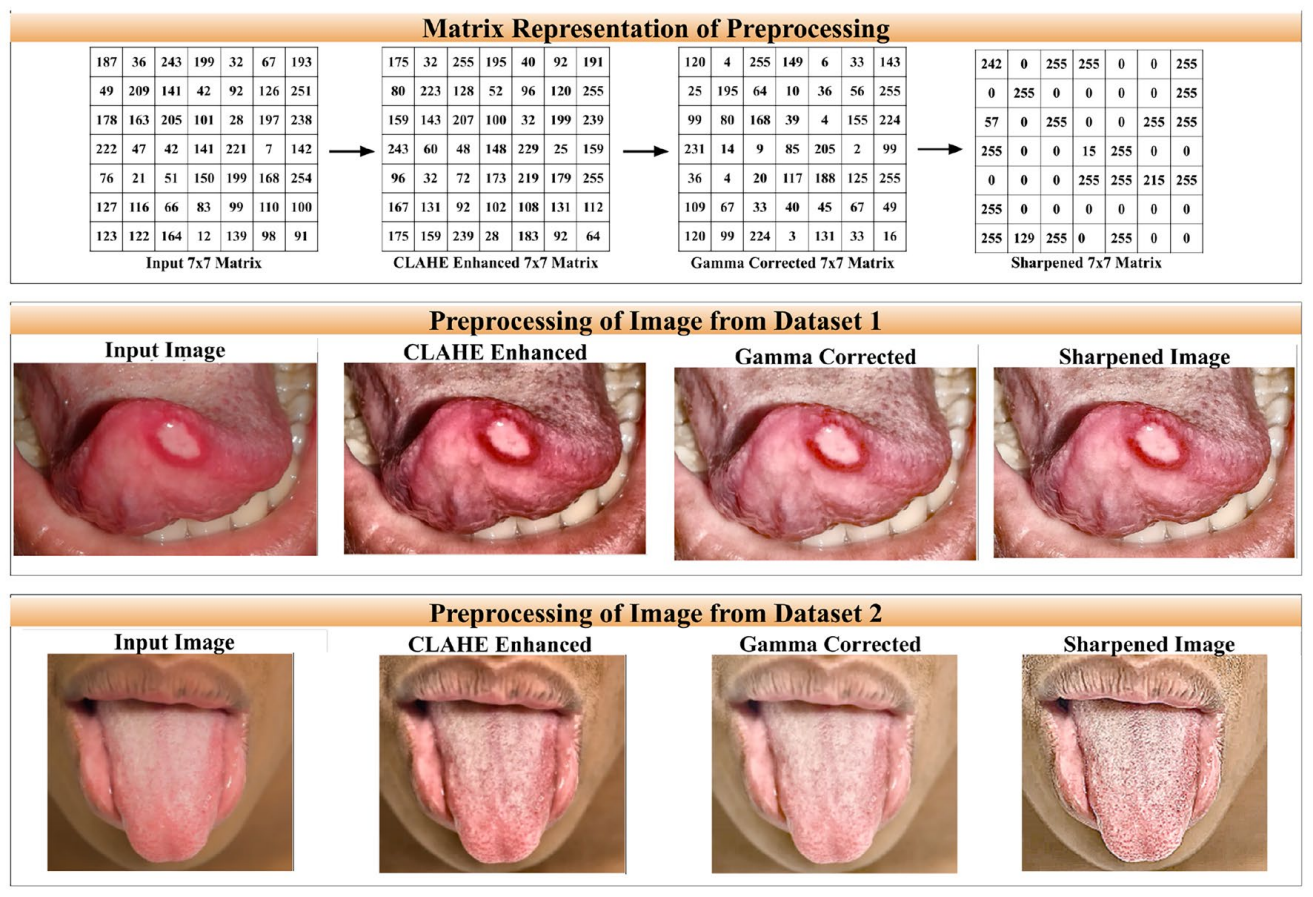
Pre-processing of oral cancer images: ROW1(from left to right): Sample pixel intensity values from image of dataset 1 and the corresponding updated values whenever a corresponding pre-processing step (as per rows below) are applied. ROW2 (from left to right): Pre-processing of an image from dataset 1 as per the steps given in Sect. Preprocessing. ROW3 (from left to right): Pre-processing of an image from dataset 2 as per the steps given in Sect. Preprocessing.

The various steps and corresponding changes in pixel intensities for images of two datasets are given in Fig. 1.

To assess robustness, we experimentally evaluated multiple parameter settings and different permutations of the preprocessing steps. Among the tested combinations, the proposed sequence, with the selected parameter values produced the best validation performance, and was therefore adopted in this work (Table 3).

**Table 3:** Key preprocessing hyperparameters used in this study.

Method	Hyperparameter(s)
CLAHE	clipLimit = 2.0
Gamma correction	$\gamma$ = 0.4
Image sharpening (Laplacian)	order = 2, sharpen_strength = 1.0

### Feature extraction

#### Adaptive multi-scale local mesh ternary pattern

Medical images, especially those of cancerous lesions, often exhibit complex textures that traditional binary patterns like LBP^37^ fail to fully capture. Thereby in the proposed approach, an Adaptive Multi-Scale Local Mesh Ternary Patterns (AM-LMTP) are extracted from the preprocessed images. AM-LMTP is a texture feature extraction method that quantifies image texture by first calculating local mesh ternary patterns^38^. A modified version of LMTP is computed by using an adaptive threshold and concatenating multi scale pattern descriptors for subsequent classification tasks. Following are the steps followed in the calculation of the AM-LMTP:

*Computation of LTP and threshold* Firstly, Local Ternary Patterns(LTP) are computed as follows:

3 $$\begin{aligned} \text {LTP}_a(P, R) = \sum _{i=1}^{P} P_i \cdot \phi _1(\gamma _{\beta |R} - \gamma _{i|R}) \end{aligned},$$

with $P$ as the number of neighboring pixels, $R$ as the radius, $a$ as the index for the order of the derivative, $P_i$ representing the pixel intensity of the $i$-th neighbor, $\gamma _{\beta |R}$ and $\gamma _{i|R}$ as the pixel intensities of the $\beta$-th and $i$-th neighbors at radius $R$, respectively, $\phi _1(x)$ computing the dissimilarity between $\gamma _{\beta |R}$ and $\gamma _{i|R}$, defined as:

4 $$\begin{aligned} \phi _1(x) = {\left\{ \begin{array}{ll} 1 & \text {if } x \ge 0 \\ 0 & \text {otherwise} \end{array}\right. } \end{aligned},$$

$\beta$ is calculated as

5 $$\begin{aligned} \beta = 1 + \text {mod}((i + P + a - 1), P) \end{aligned},$$

Then an adaptive threshold is computed as follows:

6 $$\begin{aligned} T_{adaptive}(x, y) = \alpha \cdot \sigma _{(x, y)} + \beta \end{aligned},$$

Here, $T_{adaptive}(x, y)$ is the adaptive threshold at coordinates (x,y), $\alpha$ is a scaling factor (control parameter), $\sigma _{(x, y)}$ is the local standard deviation of the neighborhood centered at (x,y) and $\beta$ is an offset or bias (control parameter).

**Fig. 2:**
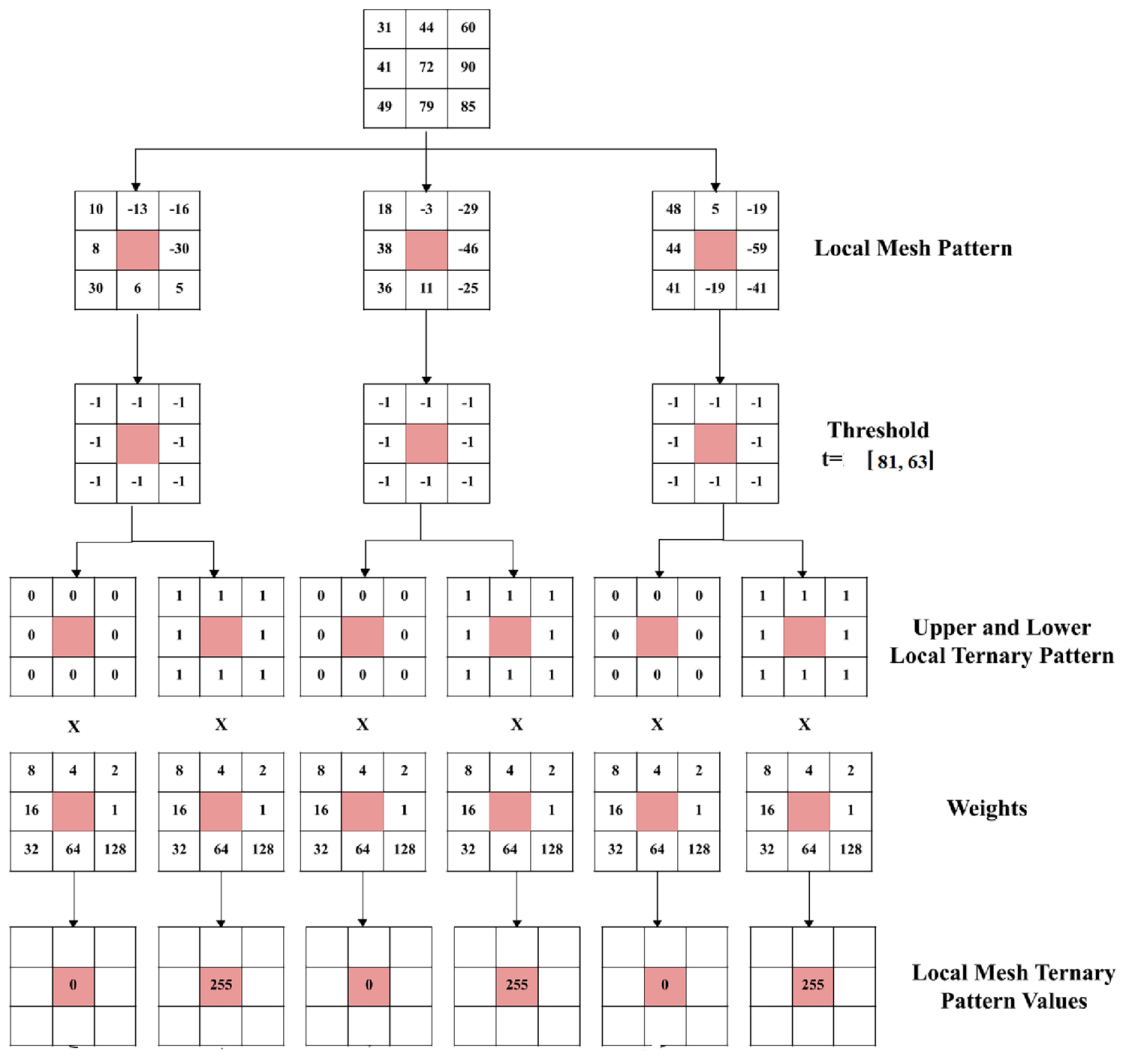
Final computation of enhanced dynamic local mesh ternary pattern from a 3x3 matrix (extracted in Fig. 1).

For e.g. in the 3x3 matrix given in Fig. 2, the adaptive threshold can be calulated as follows:

(i) Variance for the values given in 3x3 matrix = 399.74 = 400 (approx)

(ii) Standard Deviation = 20

(iii) If $\alpha$ = 0.2 and $\beta$ = 5, using Eq. (6), Adaptive threshold is computed as follows:

$$T_{adaptive}(x, y)$$ = 0.2*20+5 = 9

So final thresholds will be

(iv) Pixel Intensity at Center + Adaptive Threshold = 72+9 = 81

(v) Pixel Intensity at Center - Adaptive Threshold = 72-9 = 63

*Significance of  *$\alpha$  and  $\beta$:

High $\alpha$ increases the threshold, making the descriptor to encode a pixel difference as a significant change (1 or $-1$) if the difference is very large relative to the local contrast. This is useful in high-noise environments where random fluctuations are prominent. Low $\alpha$ makes the descriptor more sensitive to small but important texture changes whereas $\beta$ acts as a “floor” for the threshold. $\beta$ prevents the threshold from becoming 0 in perfectly flat or homogeneous regions. If $\sigma _{(x,y)}$ is zero, $T_{adaptive}$ would be zero without $\beta$. $\beta$ is usually chosen based on the noise floor of the imaging modality. For 8-bit images (0-255), a $\beta$ between 3 and 10 is common to ensure that “near-identical” pixels are grouped together as 0.

In the proposed work, the value of $\alpha$ as 0.2 suppressed noise, while $\beta = 5$ acted as an effective bias to stabilize the threshold in homogeneous regions of the biomedical images. An ablation study with different values of $\alpha$ and $\beta$ is given in Table 3 to justify this (Table 4).

**Table 4:** Comparison of different values of $\alpha$ and $\beta$ for random forest classifier on the proposed feature vector.

$\alpha$	$\beta$	Accuracy (dataset 1)	Accuracy (dataset 2)
0.1	1	0.9797	0.9823
0.2	4.5	.9810	0.9934
**0.2**	**5**	**0.9829**	**0.9989**
0.3	5.5	0.9755	0.9889
0.4	5.5	0.9754	0.9889
0.4	6	0.9734	0.9880

Significant values are in bold.

*Computation of upper and lower local ternary patterns:*

Thereby, the Upper Local Mesh Ternary Pattern(Up_LTP) and Lower Local Mesh Ternary Pattern(Low_LTP) are computed as follows:

7 $$\begin{aligned} & \text {Up\_LTP}_{ia} = {\left\{ \begin{array}{ll} 1 & \text {if } \text {LMP}_{ia} \ge 0 \\ 0 & \text {otherwise} \end{array}\right. } \end{aligned}$$

8 $$\begin{aligned} & \text {Low\_LTP}_{ia} = {\left\{ \begin{array}{ll} 1 & \text {if } \text {LMP}_{ia} < 0 \\ 0 & \text {otherwise} \end{array}\right. } \end{aligned}$$

*Computation of local mesh ternary patterns:*

After obtaining the Up_LTP and Low_LTP matrices, the weights are applied to compute six values of the local ternary mesh pattern. Various extracted Enhanced LMTP features for the images of two datasets are shown in Fig. 2.

9 $$\begin{aligned} LMTP_{final} = \frac{1}{6} \sum _{i=1}^{6} LMTP_i, \end{aligned}$$

where $\hbox {LMTP}_i$ refers to the six computed local mesh ternary pattern values for the pixel in question.

*Computation of local mesh ternary patterns for 7x7 image segments*

**Fig. 3:**
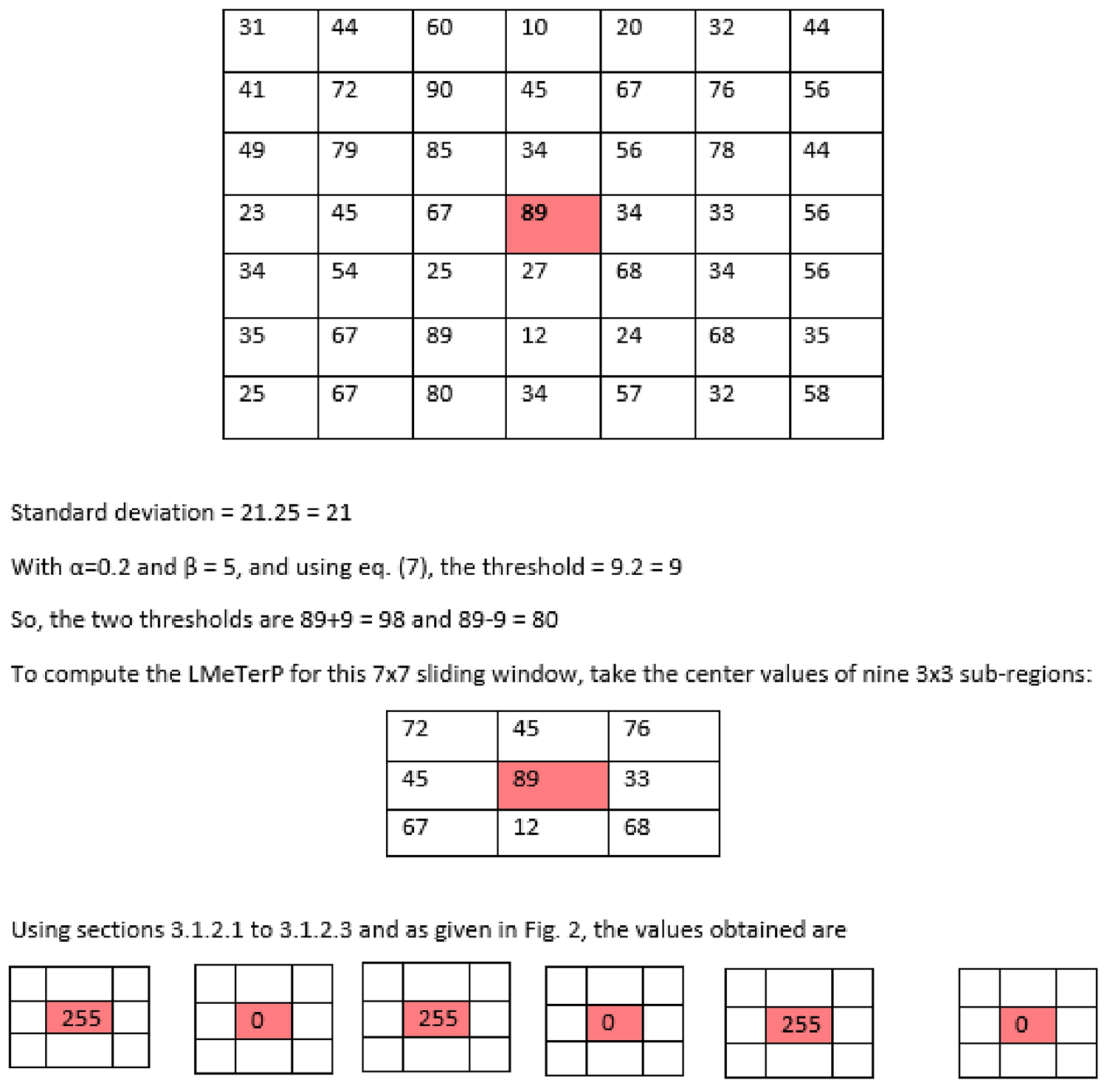
Computation of enhanced dynamic local mesh ternary pattern from a 7x7 matrix.

Similarly, computation of adaptive LMeTerP from 7x7 image segments are done. An illustration is shown in Fig. 3. All steps remains the same as given in Sect. "Computation of LTP and threshold" to Sect. "Computation of local mesh ternary patterns" except that here a 7x7 image segment is used instead of a 3x3.

**Fig. 4:**
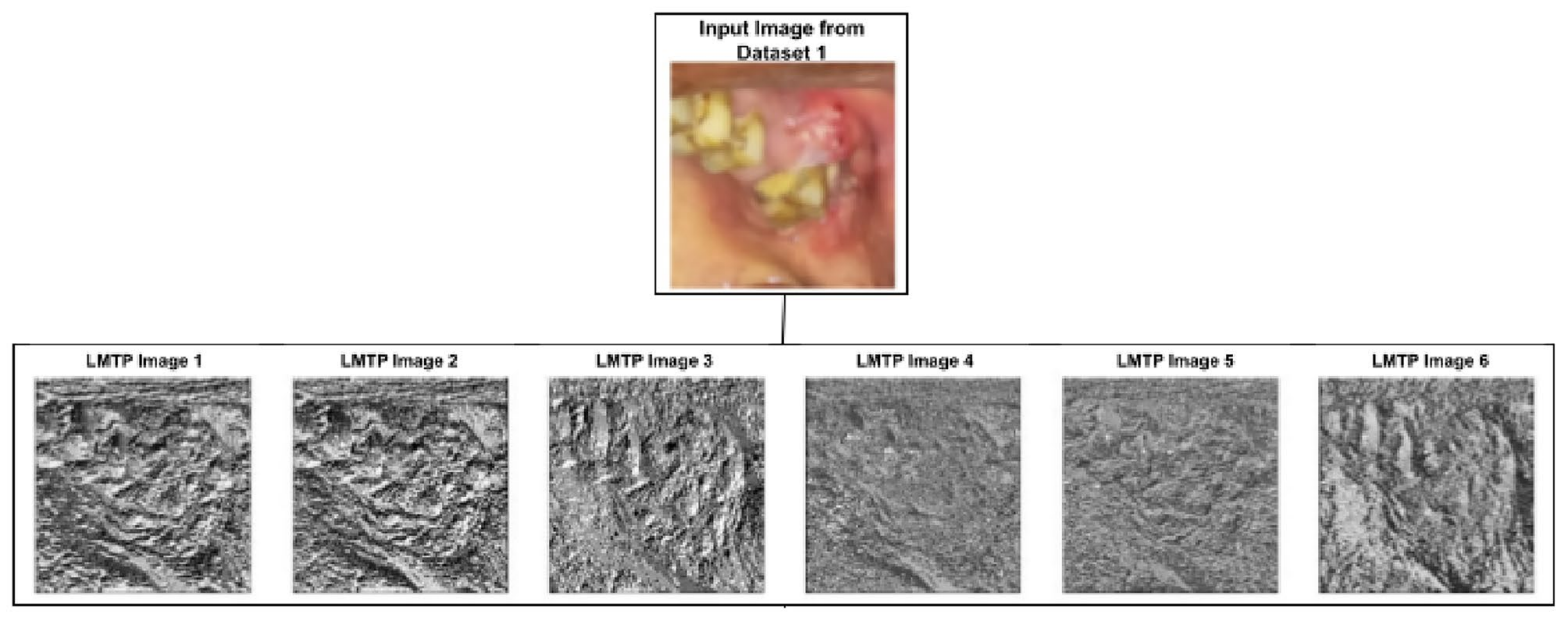
Visualization of local mesh ternary pattern (LMTP) texture images for dataset 1 for 3x3 sliding window.

**Fig. 5:**
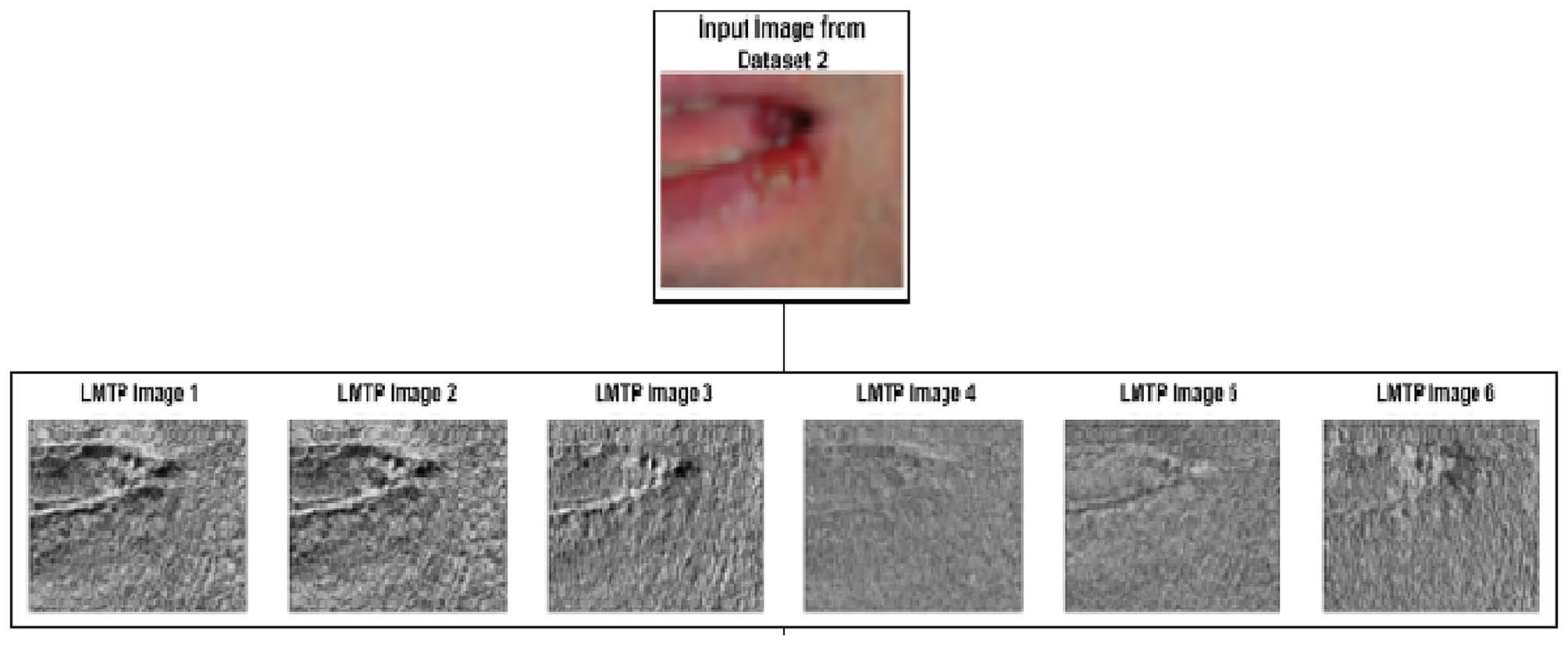
Visualization of local mesh ternary pattern (LMTP) texture images for dataset 2 for 7x7 sliding window.

The Adaptive Multiscale Local Mesh Ternary Pattern (Modified LMTP) significantly improves traditional LMeTerP by introducing an adaptive threshold that can extract the patterns while significantly highlighting the images. Also, the multiple scaling is used to highlight textures are small local scale as well as broader scale as shown in Figs. 4 and 5.

#### Histogram features

The histograms of all 3x3 sliding window-based Adaptive LMeTerP and 7x7 sliding window-based LMeTerP patterns are then computed and concatenated to obtain the final feature vector^39^. The features thereby obtained are fed into the ML models as given in the next section. For two scales, (3x3 and 7x7) and an 256-bin histogram, the final feature vector has a dimensionality of 6x512x1 (considering there are six images obtained).

### Justification for the feature vector

The feature descriptor has been modified to incorporate an adaptive threshold and also uses the feature calculated at multiple scales. The use of an adaptive threshold makes the feature vector more robust to noise, and by using multi-scale approach, both local details as well as broader textural structures are computed. The original Local Mesh ternary patterns are dependent on a fixed threshold value to deviate center pixel value. So, in homogeneous (or flat) areas of an image, any tiny amount of noise can exceed that small fixed threshold, causing the ternary code to change from a “0” to a “1” or “-1”. This may create a false pattern that can affect the final classification significantly. In the proposed formula to compute the threshold, for the flat, noisy areas, the local standard deviation ($\sigma _{(x,y)}$)is small. This will make $T_{adaptive}(x,y)$ very small. So the tiny variations in flat areas will be considered as meaningful similarities rather than noise. Similarly, in textured, high-contrast areas where edges exist, the local standard deviation ($\sigma _{(x,y)}$) is large. This makes $T_{adaptive}(x,y)$ large. This will result in only encoding a difference as non-zero (“1” or “-1”) if it is truly significantly different, relative to the strong local texture. Further, the use of multi-scale feature vector results in the computation of very fine microscopic details when the window is small and broader patterns when the window is larger. Thereby, the obtained feature vector enhances the original texture feature to a very advanced and powerful feature descriptor, computing image details at multiple levels and also a feature vector that can handle noise.

A paired *t*-test has been conducted to evaluate the statistical significance of the performance improvement obtained using Grey Wolf Optimization. For Dataset-1, the improvement was statistically significant ($t(5) = 4.52$, $p = 0.006$). Similarly, for Dataset-2, the improvement was highly significant ($t(5) = 5.45$, $p = 0.0028$). These results confirm that the incorporation of GWO significantly enhances classification performance.

To improve clarity and reproducibility, the complete AMLMTP procedure is summarized in Algorithm 1. The pseudocode provides a step-by-step implementation description, including central LMTP computation, adaptive threshold estimation, ternary encoding, and multiscale histogram concatenation.

**Algorithm 1:**
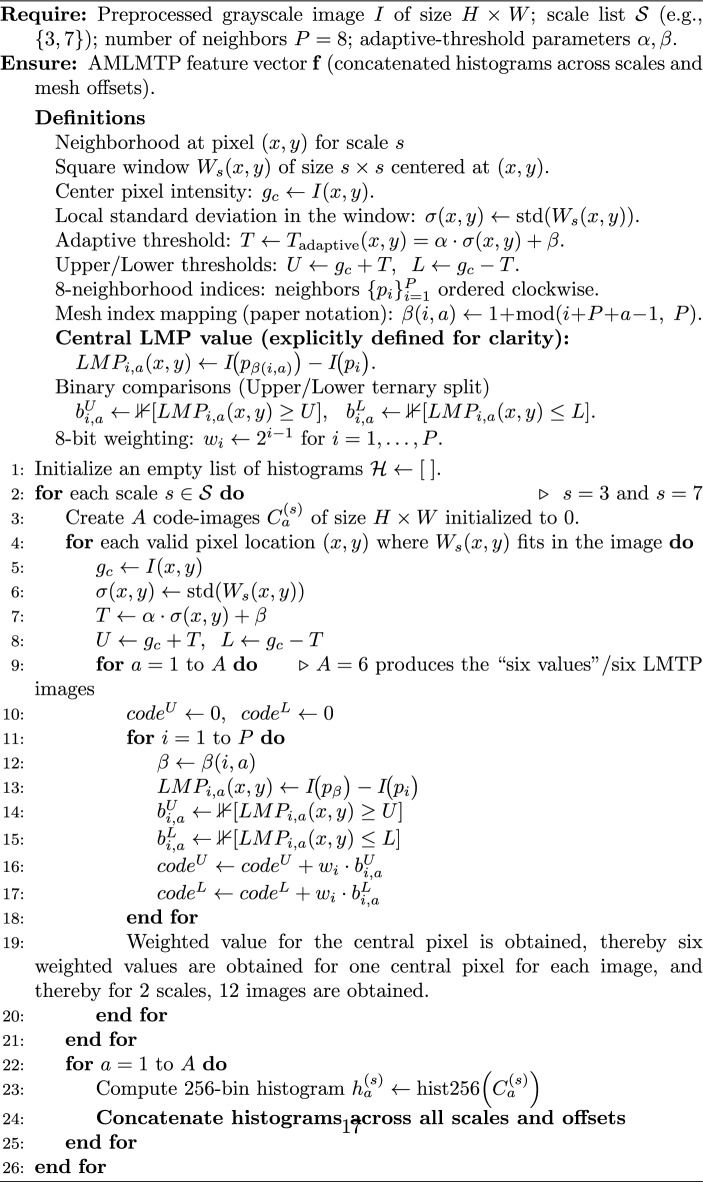
Adaptive multiscale local mesh ternary pattern (AMLMTP) feature extraction.

### Classification

In this section, the machine learning models used for the classification of oral cancer images are outlined. Each model utilizes different algorithms and techniques to accurately distinguish between different categories of oral cancer images.

#### Artificial neural network

The first network used is that of a simple Artificial Neural Network, consisting of an input layer, 10 hidden layers, and an output layer, used to learn complex patterns in the data. It adjusts weights through backpropagation to minimize the loss function, allowing for accurate classification.

10 $$\begin{aligned} h^{(j)}&= \phi (V^{(j)}h^{(j-1)} + c^{(j)}) \end{aligned},$$

where $h^{(j)}$ is the activation of the $j$-th layer, $V^{(j)}$ and $c^{(j)}$ are the weights and biases of the $j$-th layer, $\phi$ is the activation function.

#### Random forest classifier

The Random Forest algorithm constructs multiple decision trees during training and outputs the mode of the classes for classification tasks.

11 $$\begin{aligned} {\hat{z}} = \text {majority\_vote}(g_1(y), g_2(y), \ldots , g_m(y)) \end{aligned},$$

where $g_i(y)$ is the prediction of the $i$-th decision tree.

#### Gradient boosting classifier

Gradient Boosting is an ensemble technique that builds models sequentially, with each new model correcting the errors made by previous ones. It combines the strengths of multiple weak learners to create a robust predictive model.

12 $$\begin{aligned} G_{k}(y) = G_{k-1}(y) + \eta \cdot h_k(y) \end{aligned},$$

where $G_{k}(y)$ is the model at stage $k$, $G_{k-1}(y)$ is the model at stage $k-1$, $\eta$ is the learning rate, and $h_k(y)$ is the $k$-th weak learner.

#### k-nearest neighbors (KNN) classifier

The k-Nearest Neighbors algorithm classifies data points based on the majority class among its $k$ nearest neighbors in the feature space. It is simple yet effective, relying on distance metrics to make predictions.

13 $$\begin{aligned} {\hat{z}} = \text {mode}(z_{k}) \end{aligned},$$

where $z_k$ are the labels of the $k$ nearest neighbors.

#### Support vector classifier

Support Vector Classifier (SVC) constructs a hyperplane or set of hyperplane in a high-dimensional space to classify data points. It aims to find the optimal hyperplane that maximizes the margin between different classes.

14 $$\begin{aligned} g(y) = \sum _{i=1}^{m} \beta _i z_i J(y_i, y) + d \end{aligned},$$

where $\beta _i$ are the support vector coefficients, $z_i$ are the class labels, $J$ is the kernel function, and $d$ is the bias term.

### Optimization of classification models using Grey Wolf optimization

**Fig. 6:**
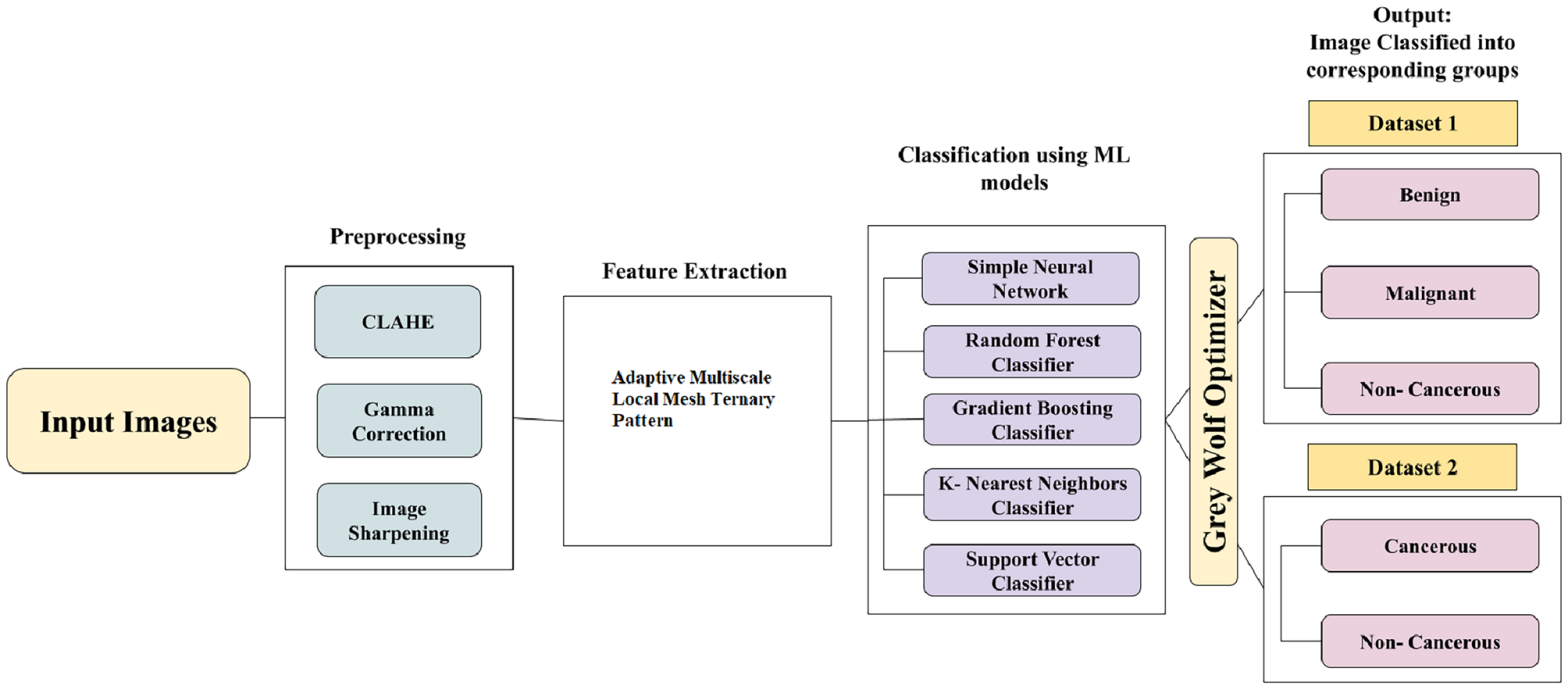
Flowchart illustrating the proposed methodology for the classification of oral cancer images.

To improve the performance of classification models, the Gray Wolf Optimization (GWO) algorithm is applied. It is a metaheuristic optimization method inspired by the social hierarchy and hunting techniques of gray wolves in the wild. It is used to fine-tune the hyperparameters of the classifiers, thereby enhancing the accuracy and robustness in classifying oral cancer images. The general methodology is described in Fig. 6.

In GWO, the top candidate solution is labeled “alpha,” followed by “beta” and “delta,” while all other solutions are termed “omega.” The algorithm adjusts the search agents (wolves) toward the best possible solution. The position update rule is described as:

15 $$\begin{aligned} {\textbf{P}}(t+1) = {\textbf{P}}_{\alpha }(t) - B \cdot E \end{aligned},$$

where ${\textbf{P}}(t+1)$ is the updated position, ${\textbf{P}}_{\alpha }(t)$ is the position of the best current solution (alpha wolf), $B$ is the coefficient that controls convergence, and $E$ is the distance between the search agent and the target solution (prey).

In the proposed work, Gray Wolf Optimization (GWO) is applied to enhance the performance of machine learning models, like simple neural networks, the random forest, the gradient boost, the k-nearest Neighbors, and the support vector classifier.

GWO dynamically explores the search space and adjusts hyperparameters based on a predefined fitness function. In this study, **classification accuracy** computed on the validation data is used as the fitness function during optimization. Accuracy was selected because it provides a direct and intuitive measure of overall classification performance across both datasets. Different classifiers have effectively utilized the Grey Wolf Optimizer (GWO) for performance enhancement through parameter optimization. In the case of a Simple Neural Network, parameters such as the number of neurons, learning rate, and batch size are fine-tuned using GWO to identify the best combination that yields optimal performance. For the Random Forest classifier, GWO dynamically adjusts hyperparameters such as the number of trees, maximum tree depth, and minimum samples required for node splitting, thereby enabling the construction of a robust ensemble of decision trees that ensures accurate classification. Similarly, in Gradient Boosting (GB), parameters like the learning rate, number of estimators, and maximum depth are optimized by GWO, leading to improved classification accuracy and reduced prediction errors. In the case of k-Nearest Neighbors (KNN), GWO optimizes crucial parameters such as the number of neighbors and the distance metric, maintaining a balance between bias and variance to achieve precise classification. Lastly, for the Support Vector Classifier (SVC), GWO fine-tunes key parameters including the regularization constant and kernel function, ensuring the model identifies the optimal hyperplane that effectively separates classes while minimizing misclassification.

By applying Grey Wolf Optimization to Simple Neural Network, Random Forest, Gradient Boosting, k-nearest Neighbors, and Support Vector Classifier models, this study achieves better classification results. The GWO algorithm’s ability to optimize hyperparameters dynamically contributes to improved accuracy and reduced error rates in oral cancer detection, offering a more precise tool for medical diagnostics.

Grey Wolf Optimization was selected because hyperparameter tuning in this framework involves nonlinear and mixed discrete–continuous search spaces where gradient-based methods are not applicable. Compared to exhaustive Grid Search, which becomes computationally expensive as the search space grows, GWO provides a balanced exploration–exploitation mechanism that requires fewer evaluations. Unlike Bayesian Optimization, GWO does not rely on surrogate modeling assumptions and remains effective in irregular objective landscapes. These characteristics make GWO suitable for efficiently optimizing classifier parameters in the proposed AMLMTP-based framework.

## Results

Different machine learning models are used on the extracted features and varying degrees of accuracy are obtained, highlighting the performance of each model in classifying or diagnosing oral cancer-related patterns within the imaging data.

### Dataset

In the present work, two distinct datasets were utilized to evaluate the proposed methodology. The first dataset consists of color images of oral lesions captured via mobile and intra-oral cameras, categorized into benign, malignant^40^, and non-cancerous. The inclusion of the non-cancerous category expands the dataset’s scope to encompass images representing non-cancerous oral conditions, thereby enhancing its diversity and utility for training and validating diagnostic algorithms. The second dataset consists of images of lips and tongue^41^, meticulously classified into cancerous and non-cancerous groups. Table 5 gives the details of the dataset used in the study.

Because Dataset-1 (573 images) and Dataset-2 (131 images) are relatively small, we control overfitting using **stratified** *k***-fold cross-validation** and report results as **mean±std** across folds. Hyperparameter tuning is performed only on the training folds to prevent data leakage. In addition, **L1 regularization** (for linear models where applicable) and capacity controls are applied to reduce memorization and improve generalization. To address class imbalance in datasets, **image augmentation** is applied exclusively to the training data within each fold, increasing minority-class representation through controlled transformations. Dataset 1 contains 573 original images, we generated two augmented variants per original (1,146 augmented images), yielding 1,719 images in total. Dataset 2 contains 131 original images, similarly, we generated 262 augmented images, yielding 393 images in total. For both datasets, we used an 80:20 stratified train–test split, and augmentation was applied only to the training set to avoid data leakage. To prevent data leakage, it has been ensured that no duplicate or near-duplicate images appear across training and validation folds.

**Table 5:** Details of the datasets used.

Dataset attribute	Dataset 1	Dataset 2
Total number of images	573	393
Image Formats	JPG, JPEG	JPG, JPEG, PNG
Number of Classes	3	2
Class Types	Benign, Malignant, Non-Cancer	Cancer, Non-Cancer

### Evaluation metrics

The key metrics used in this study include precision, recall, accuracy, and the Receiver Operating Characteristic (ROC) curve^42,43^.

Precision is the ratio of true positive instances among all instances predicted as positive. The formula is given below:

16 $$\begin{aligned} {P_r} = \frac{{T_{Pos}}}{{T_{Pos}} + {F_{Pos}}} \end{aligned},$$

where $P_r$ denotes Precision, $T_{Pos}$ is True Positives, and $F_{Pos}$ is False Positives.

Recall, also known as sensitivity, quantifies the proportion of true positive instances that were correctly classified. The formula for recall is:

17 $$\begin{aligned} {R_c} = \frac{T_{Pos}}{T_{Pos} + F_{Neg}} \end{aligned},$$

where $R_c$ is Recall, $T_{Pos}$ is True Positives and $F_{Neg}$ is False Negatives.

Accuracy represents the overall correctness of the classifier’s predictions and is calculated as

18 $$\begin{aligned} {A_c} = \frac{{N_{CP}}}{{T_{NP}}} \times 100 \% \end{aligned},$$

where $A_c$ is accuracy, $N_{CP}$ denotes correct Predictions and $T_{NP}$ denotes total number of predictions.

The ROC curve shows the classifier’s performance across different classification thresholds regarding the true positive rate (sensitivity) against the false positive rate (1 - specificity). The true positive rate ($T_{PR}$) and false positive rate ($F_{PR}$) are computed as follows:

19 $$\begin{aligned} & {T_{PR}} = \frac{T_{Pos}}{T_{Pos} + F_{Neg}} \end{aligned},$$

20 $$\begin{aligned} & {F_{PR}} = \frac{F_{Pos}}{{F_{Pos}} + {T_{Neg}}} \end{aligned},$$

The result analysis will provide in-depth information about the models’ precision, recall, and accuracy, resulting in a comprehensive assessment of accurate oral cancer diagnosis.

### Experiment-1 using random forest classifier (RFC)

**Fig. 7:**
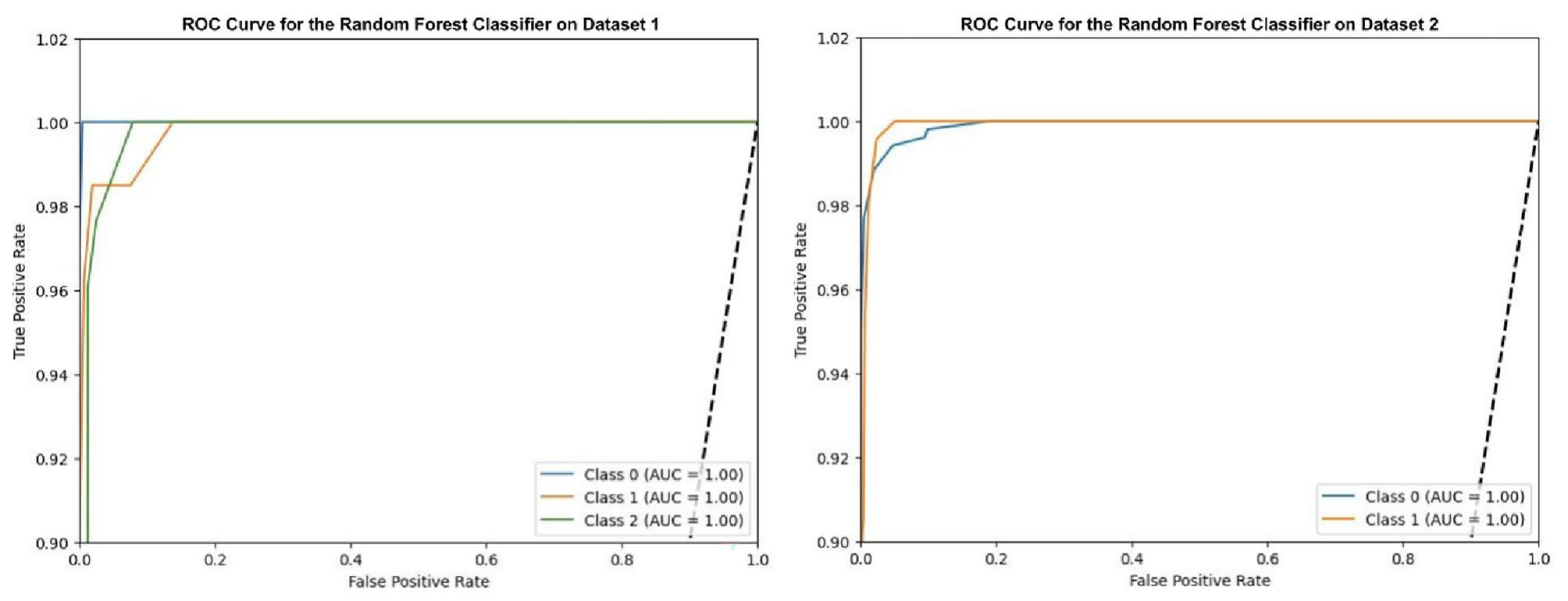
ROC curves for optimized random forest classifier.

In this case, the RFC is optimized with Grey Wolf Optimization (GWO) and applied to the oral cancer image datasets as shown in Fig 7. After optimization, the RFC shows excellent performance with an accuracy of 98.29% on Dataset-1 and 99.89% on Dataset-2. The results show that the basic random forest model achieves an accuracy of 97.29% on Dataset-1 and 98.24% on Dataset-2. This proves a significant improvement in post-optimisation.

### Experiment-2 using gradient boosting classifier (GBC)

**Fig. 8:**
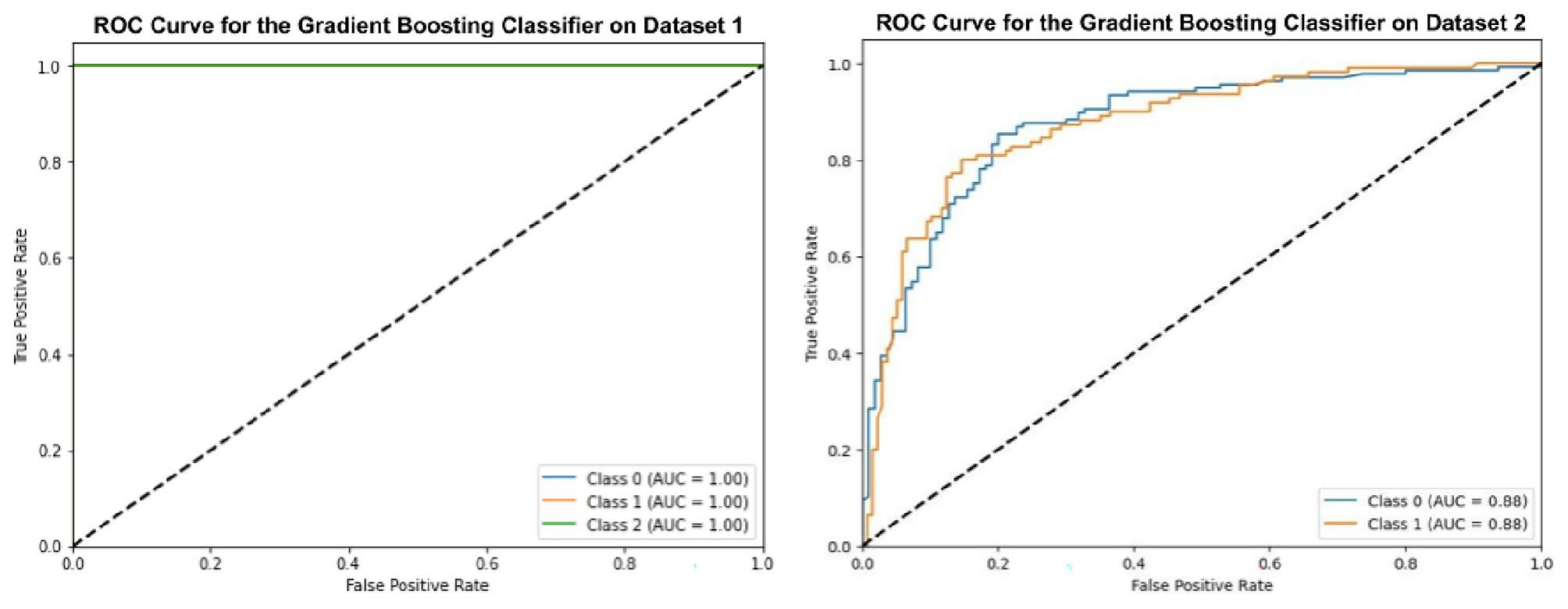
ROC curves for optimized gradient boosting classifier.

In this scenario, the GBC is optimized using the GWO algorithm and applied to the oral cancer image datasets as shown in Fig. 8. Similar to Experiment 1, after optimization, this method shows an accuracy of 99.66% on Dataset-1 and 80.56% on Dataset-2. In comparison, in the basic model without optimization, the accuracy is 98.66% for Dataset-1 and 79.56% for Dataset-2. This shows a clear improvement with optimization. The analysis proves that the GBC outperforms most other models, but it has not achieved the same level of accuracy as the optimized RBC.

### Experiment-3 using K-nearest neighbors (KNN) classifier

**Fig. 9:**
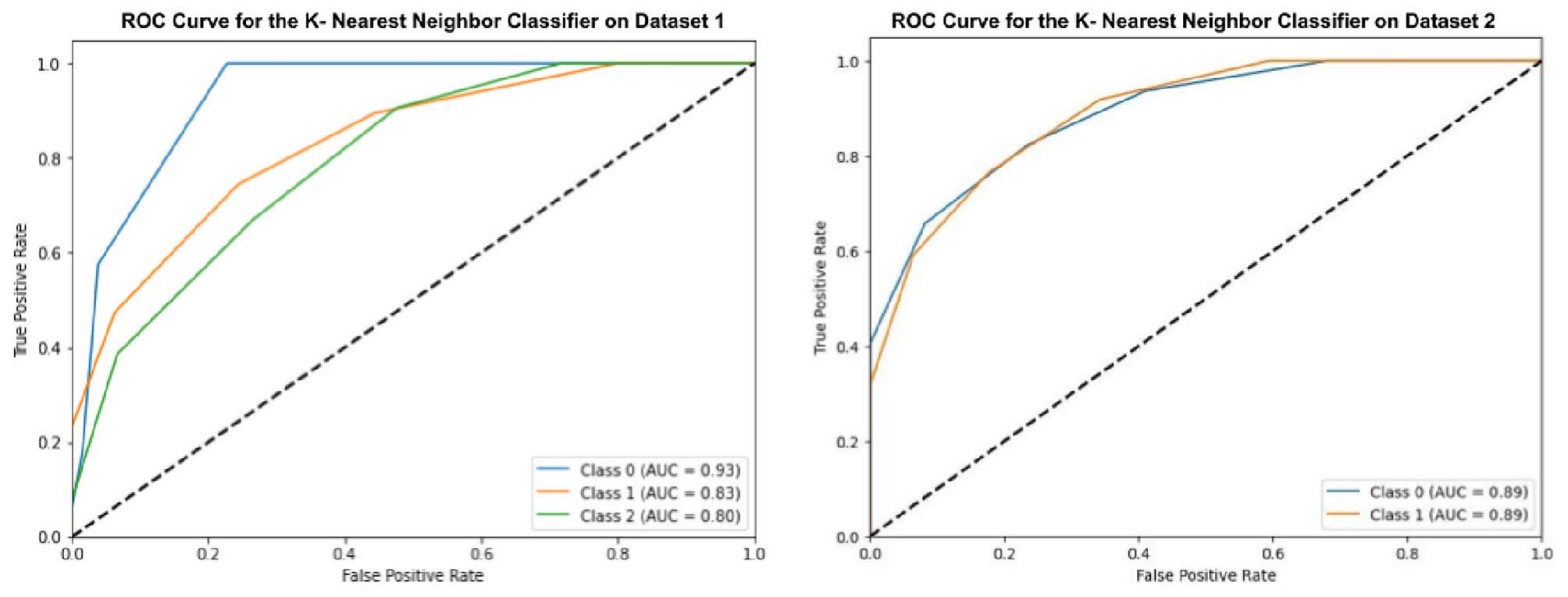
ROC curves for optimized K-nearest neighbors classifier.

The KNN classifier is also optimized with GWO, and it is applied to the oral cancer image datasets as shown in Fig. 9. The optimized KNN achieves an accuracy of 67.24% on Dataset-1 and 79.57% on Dataset-2. The basic KNN model achieved slightly lower accuracies of 63.24% on Dataset-1 and 75.57% on Dataset-2.

The analysis proves that KNN performs reasonably well on Dataset-2, it has not matched the performance of the gradient boosting or random forest classifiers.

### Experiment 4 using simple neural network

**Fig. 10:**
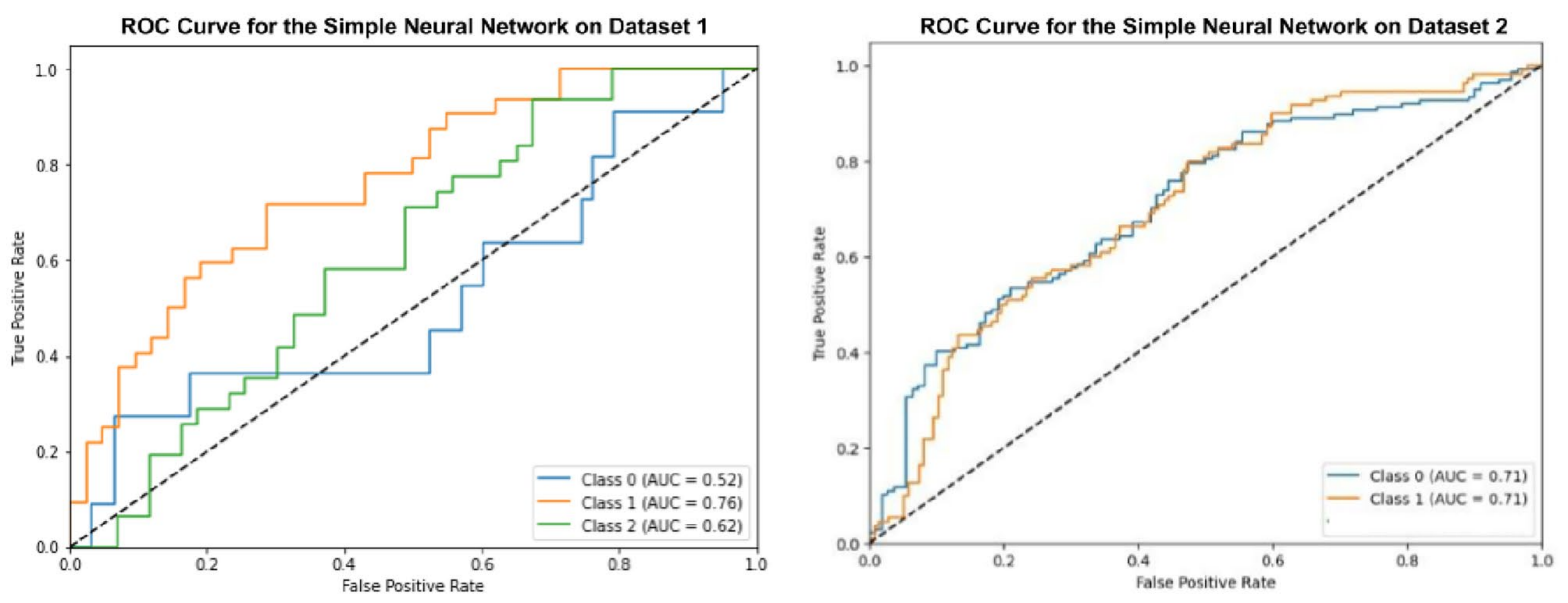
ROC curves for optimized simple neural network.

Here an Artificial Neural Network, optimized using Grey Wolf Optimization, has been employed for the classification of the oral cancer images as shown in Fig. 10. The optimized Simple Neural Network achieved an accuracy of 47.9% on Dataset 1 and 66.39% on Dataset 2. In contrast, the basic model without optimization underperformed, with 45.90% on Dataset 1 and 61.39% on Dataset 2. The Simple Neural Network performed adequately, but did not match the accuracy levels of the other models.

### Experiment 5 using support vector classifier

**Fig. 11:**
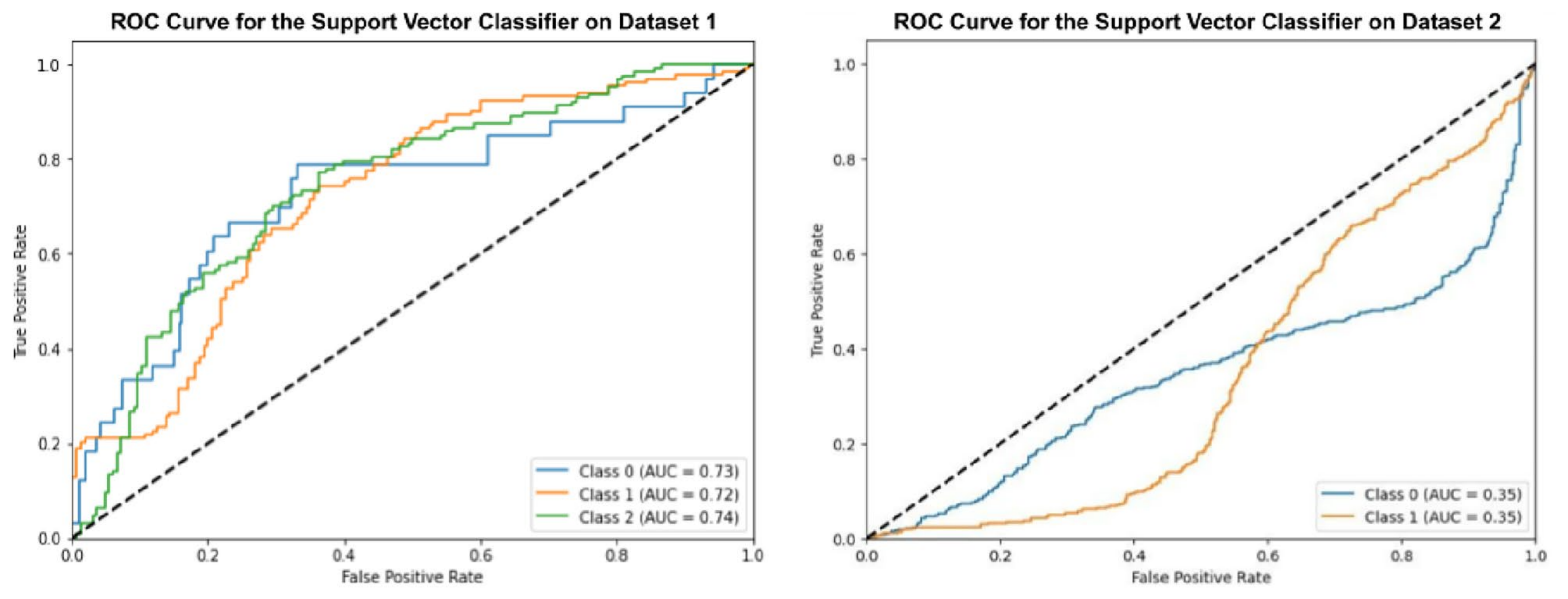
ROC curves for optimized support vector classifier.

In this section, the Support Vector Classifier (SVC), optimized using Grey Wolf Optimization, was applied to the oral cancer image datasets as shown in Fig. 11. The optimized SVC achieved an accuracy of 50.17% on Dataset 1 and 52.64% on Dataset 2. The basic SVC model achieved slightly lower accuracies of 47.17% on Dataset 1 and 48.64% on Dataset 2. The SVC showed moderate performance, but did not achieve the same accuracy as the other models.

### Experiment 6 using convolutional neural network

**Fig. 12:**
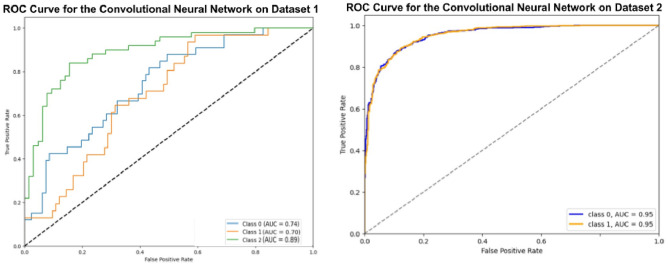
ROC curves for optimized convolutional neural network.

In this section, the Convolutional Neural Network (CNN), optimized using Grey Wolf Optimization, was applied to the oral cancer image datasets as hown in Fig. 12. The optimized CNN model achieved an accuracy of 90.00% on Dataset 1 and 82.77% on Dataset 2. In comparison, the basic CNN model reached 88.00% on Dataset 1 and 78.77% on Dataset 2. While the CNN demonstrated strong performance, particularly on Dataset 1, it did not surpass the optimized Random Forest Classifier’s accuracy on Dataset 2. The detailed results for precision and recall of different classifiers along with Grey Wolf Optimizer for dataset 1 are given in Table 6 and 7 and the corresponding results for dataset 2 are given in Table 8 and 9. Also, the final accuracy is summarized for both the datasets in Table 10.

**Table 6:** Comparison of ML classifiers on the basis of precision with grey Wolf optimization on dataset 1 for different output classes.

Classifier with optimization	Precision		
	Class 0 (Non Cancer)	Class 1 (Benign)	Class 2 (Malignant)
Random forest classifier	1.00	0.96	1.00
Gradient boosting classifier	1.00	0.99	1.00
KNN classifier	0.68	0.68	0.66
Simple neural network	0.65	0.60	0.71
Support vector classifier	0.00	0.65	0.48
Convolutional neural network	0.65	0.87	0.91

**Table 7:** Comparison of ML classifiers on the basis of Recall with Grey Wolf Optimization on Dataset 1 for different output classes.

Classifier with optimization	Recall		
	Class 0 (Non Cancer)	Class 1 (Benign)	Class 2 (Malignant)
Random forest classifier	0.97	1.00	0.97
Gradient boosting classifier	1.00	1.00	0.99
KNN classifier	0.39	0.74	0.67
Simple neural network	0.52	0.80	0.50
Support vector classifier	0.00	0.21	0.94
Convolutional neural network	0.82	0.75	0.81

**Table 8:** Comparison of ML classifiers on the basis of Precision with Grey Wolf Optimization on Dataset 2 for different output classes.

Classifier with optimization	Class 0 (cancer)	Class 1 (non cancer)
Random forest classifier	1.00	0.98
Gradient boosting classifier	0.92	0.88
KNN classifier	0.79	0.80
Simple neural network	0.68	0.64
Support vector classifier	0.53	1.00
Convolutional neural network	0.89	0.79

**Table 9:** Comparison of ML classifiers on the basis of Recall with Grey Wolf Optimization on Dataset 2 for different output classes.

Classifier with optimization	Class 0 (cancer)	Class 1 (non cancer)
Random forest classifier	0.98	1.00
Gradient boosting classifier	0.89	0.91
KNN classifier	0.82	0.76
Simple neural network	0.68	0.65
Support vector classifier	1.00	0.00
Convolutional neural network	0.72	0.93

**Table 10:** Accuracy of different Machine learning based classifiers with and without Grey Wolf Optimization on Dataset 1 and Dataset 2.

**Classifiers**	Without Grey Wolf optimization		With Grey Wolf optimization	
	Dataset 1 accuracy	Dataset 2 accuracy	Dataset 1 accuracy	Dataset 2 accuracy
Random forest classifier	97.29%	98.24%	98.29%	99.89%
Gradient boosting classifier	98.66%	79.56%	99.66%	80.56%
KNN classifier	63.24%	75.57%	67.24%	79.57%
Simple neural network	45.90%	61.39%	47.90%	66.39%
Support vector classifier	47.17%	48.64%	50.17%	52.64%
Convolutional neural network	88.00%	78.77%	90.00%	82.77%

For Dataset 1, the Random Forest Classifier shows an improvement of 46.22% over KNN, 95.92% over the Support Vector Classifier, and 105.22% over the Simple Neural Network. However, it is slightly less accurate than the Gradient Boosting Classifier by 1.38%. As for Dataset 2, the Random Forest Classifier demonstrates an improvement of 24.01% over the Gradient Boosting Classifier, 25.52% over KNN, 89.74% over the Support Vector Classifier, and 50.46% over the Simple Neural Network. These improvements highlight the effectiveness of the proposed methodology, showcasing significant enhancements in classification performance across various classifiers. To prove the superiority of the proposed method, we have compared the performance with existing solutions cited with their reference as given in Table 11. The results prove the superiority of the proposed method.

The proposed model is also compared with recent explainable AI based models in medical diagnosis, i.e. Grad-CAM-based XAI^44^. As summarized in Table 12, although the XAI models provide heatmap-based explanations, they require substantially higher computational resources (larger parameter counts and higher CPU inference time), whereas the proposed AMLMTP-based pipeline remains lightweight and computationally efficient while maintaining competitive precision and recall performance.

All timing experiments were performed on Google Colab CPU runtime (OS: Linux 6.6.113+, x86_64; Python 3.12.12; RAM: 12.67 GB; NumPy 2.0.2; Pandas 2.2.2; PyTorch 2.10.0+cpu; Torchvision 0.25.0+cpu). Runtimes in Table 12 report end-to-end wall-clock time for the stated pipeline configuration, including preprocessing, feature extraction, and model training/inference.

**Table 11:** Comparison of model performance across papers.

Model	Precision	Recall
*P*roposed model	99.80%	99.40%
*L*MTP	78.80%	78.66%
*L*BP	47.62%	47.48%
*L*ogistic regression	60.76%	61.74%
*H*RNet^32^	84.30%	83.00%
*D*enseNet121^36^	99%	100%
*F*aster R-CNN^36^	76.67%	82.14%
*C*onvNeXt + Grad-CAM^44^	83.7%	75.6%
*M*obileNet + Grad-CAM^44^	92.8%	63.4%

**Table 12:** Computational efficiency comparison between AMLMTP-based pipeline and deep-learning baselines.

Efficiency attribute	Total time
AMLMTP + Random forest classifier	2 min 11 sec
AMLMTP + Gradient boosting classifier	2 min 30 sec
AMLMTP + KNN classifier	1 min 30 sec
AMLMTP + Simple neural network	2 min 15 sec
AMLMTP + Support vector classifier	1 min 46 sec
CNN Baseline (DenseNet121^36^)	3 min 45 sec

## Conclusion

This study proposed an effective algorithm for feature extraction in oral cancer images and investigated the application of various machine learning classifiers, both in their basic and optimized forms, for classifying oral cancer patterns in smartphone-based images of lips and tongue, utilising two distinct publicly available datasets and different feature extraction techniques. The proposed methodology used an extensive preprocessing technique with CLAHE, Gamma correction and sharpening of images using Laplacian filter. Then a feature extraction is done using AM-LMTP. Here the conventional LMTP is extended by introducing an adaptive threshold and extracting features by multiscale sliding window. The study then evaluated the performance of the Random Forest Classifier, Simple Neural Network, Gradient Boosting Classifier, Support Vector Classifier, and KNN Classifier, with comprehensive experiments comparing their accuracy before and after optimization using Grey Wolf Optimization.

The results demonstrated significant improvements across most models after optimization. The Random Forest Classifier, which was already a strong performer in its basic form, achieved an accuracy of 98.29% on Dataset 1 and 99.89% on Dataset 2 in its optimized state, outperforming all other classifiers.

The comparison between traditional machine learning classifiers and Convolutional Neural Networks (CNNs) revealed that, while CNNs performed well, traditional classifiers like Random Forest and Gradient Boosting offered superior performance in terms of accuracy. Moreover, these models required less computational power and training time, making them more suitable to be deployed on handheld devices or devices that do not possess heavy computational resources. Also, the technique suits environments where real time results are to be generated.

The findings underscore the potential of Random Forest and Gradient Boosting Classifiers for accurate early detection and diagnosis of oral cancer.

While the proposed algorithm is superior to many state-of-the-art techniques, however it has some constraints as well. One of the key constraint is that a limited dataset has been used for training. The algorithm might perform differently for histopathological images. Further more ablation can be performed to tune the hyperparameters of deep networks that might perform better thereafter on the obtained features. Also in images where the critical features are on the periphery, the algorithm might not be able to detect features due to use of a 7x7 window.

## Data Availability

The datasets used in this study are publicly available
